# Plant growth promoting rhizobacteria *Dietzia natronolimnaea* modulates the expression of stress responsive genes providing protection of wheat from salinity stress

**DOI:** 10.1038/srep34768

**Published:** 2016-10-06

**Authors:** Nidhi Bharti, Shiv Shanker Pandey, Deepti Barnawal, Vikas Kumar Patel, Alok Kalra

**Affiliations:** 1 Microbial Technology Department, CSIR-Central Institute of Medicinal and Aromatic Plants, Lucknow- 226015, India

## Abstract

Plant growth promoting rhizobacteria (PGPR) hold promising future for sustainable agriculture. Here, we demonstrate a carotenoid producing halotolerant PGPR *Dietzia natronolimnaea* STR1 protecting wheat plants from salt stress by modulating the transcriptional machinery responsible for salinity tolerance in plants. The expression studies confirmed the involvement of ABA-signalling cascade, as *TaABARE* and *TaOPR1* were upregulated in PGPR inoculated plants leading to induction of *TaMYB* and *TaWRKY* expression followed by stimulation of expression of a plethora of stress related genes. Enhanced expression of *TaST*, a salt stress-induced gene, associated with promoting salinity tolerance was observed in PGPR inoculated plants in comparison to uninoculated control plants. Expression of SOS pathway related genes (*SOS1* and *SOS4*) was modulated in PGPR-applied wheat shoots and root systems. Tissue-specific responses of ion transporters *TaNHX1*, *TaHAK*, and *TaHKT1*, were observed in PGPR-inoculated plants. The enhanced gene expression of various antioxidant enzymes such as *APX*, *MnSOD*, *CAT*, *POD*, *GPX* and *GR* and higher proline content in PGPR-inoculated wheat plants contributed to increased tolerance to salinity stress. Overall, these results indicate that halotolerant PGPR-mediated salinity tolerance is a complex phenomenon that involves modulation of ABA-signalling, SOS pathway, ion transporters and antioxidant machinery.

The continually growing world population presents a focal challenge for existing agricultural practices to warranty solution to a steadily escalating demand in food production. This increasing want for food is paralleled by striking losses of arable land owing to rising severity of soil annihilation by abiotic environmental conditions. Salinity is a major environmental stress that severely affecting plant productivity worldwide. Exposure of plants to excess salt triggers imbalance of ions, ion toxicity-induced metabolism imbalances and hyperosmotic stress induced water deficit. Plants follow various kinds of salt-tolerant mechanisms like osmolytes and polyamines synthesis, reducing reactive oxygen species level by following antioxidant defense mechanism, and ions transport and their compartmentalization. Plants have stress-specific adaptive responses as well as the response that protects plants against more than one environmental stress^1^. Numerous abiotic stress-related genes, transcription factors and regulatory elements in promoters, have been characterized^2^. Plants utilize three strategies to prevent as well as adapt to high Na^+^ concentrations: (i) active Na^+^ efflux, (ii) Na^+^ influx prevention and (iii) Na^+^ compartmentalization in vacuoles^3^. Antiporters play a significant role in ion homeostasis in plants. Na^+^/H^+^ antiporters (SOS1 and NHX1) minimize the cytotoxicity by maintaining the optimal cytosolic ions concentration. NHX1, located in tonoplast reduces cytosolic Na^+^ concentration by pumping Na^+^ in to the vacuole^4^, whereas plasma membrane localized SOS1 extrudes Na^+^ in apoplasts^5^ and both of these process are driven by proton motive force generated by the H^+^ -ATPase^6^. Salinity alters the normal homeostasis of the cell and causes an increased production of reactive oxygen species (ROS)^7^. Under optimal growth conditions, antioxidant defense mechanisms of plants can cope with increased ROS level, whereas, during stress conditions, the antioxidant capacity is overcome by ROS production. Therefore, tight regulation of the steady-state concentrations of ROS by antioxidative mechanism is critical to minimize the oxidative stress-mediated damage in plant cells and consequently allowing ROS to perform useful functions as signal molecules^7^.

Plant-growth-promoting rhizobacteria (PGPR) are a group of microbes which colonize the plant roots and improve plant growth either directly or indirectly^8^. Various studies have been already published that explain the effect of PGPR in relieving abiotic stress in different crop plants^9^,^10^. PGPR promote plant growth by altering the selectivity of Na^+^, K^+^, and Ca^2+^ and sustain a higher K^+^/Na^+^ ratio in plants under salt stress^11^. With significant improvements in our understanding of PGPR-mediated salinity tolerance in host plant, efforts are being made to understand the tolerance mechanism at the gene expression level. Thus, plant cellular and molecular responses which are generally studied during interactions with various types of symbiotic as well as mutualistic microbes are of great interest, since the responses may be related to an essential process for establishing different relationships between plants and microbes. There are diverse PGPR-induced changes in plants, and growth promotion perhaps results due to a complex combination of various PGPR-induced mechanisms that affect both plant development as well as plant nutrition^12^. Furthermore, transcriptomic analyses have been conducted for only a few rhizobacterial strains to unravel the changes in molecular mechanisms in plants associated with PGPR-mediated plant growth promotion^13^,^14^. Further understanding of the primary mechanisms employed by these bacteria will likely accelerate the recognition of PGPR as appropriate and efficient adjuncts to agricultural practice.

In our earlier experiments, we identified some PGPRs capable of inducing tolerance against NaCl stress in wheat plants (data unpublished); of these *Dietzia natronolimnaea* strain, STR1 was found to provide maximum protection to the plants under salt stress. *D. natronolimnaea* strain STR1 was also found to protect *Ocimum basilicum* plant from salt stress^15^. With the aim of understanding the possible mechanisms involved in PGPR-mediated salinity tolerance in wheat plants, we studied the expression of different genes involved in salt stress tolerance in plants.

## Results

### Phylogenetic analysis and functional traits of *Dietzia natronolimnaea* strain STR1

Phylogenetic analysis confirmed that isolate STR1 (Accession no. KJ413139) has maximum similarity with *Dietzia natronolimnaea* strain CBS 107.95 (Accession no. NR_026273) (Query coverage 100%, percentage of similarity 99%, E-value 0.0) (Supplementary Fig. S1). Plant growth promoting traits like phosphate solubilisation, indole acetic acid (IAA) production, nitrogen fixation and ACC deaminase activity were found to be negative in *D. natronolimnaea* STR1. The bacterial strain was also found to be negative for exopolysaccharide production and *nifH* gene (data not shown). The growth curves at different NaCl concentrations and pH have been shown in Supplementary Fig. S2. *D. natronolimnaea* STR1 showed the best growth at 0.5 M NaCl and neutral pH (i.e. pH 7).

### Colonization of wheat plant with *Dietzia natronolimnaea* STR1

Scanning electron microscopy confirmed that the untreated control plants did not harbour any bacteria and the roots of *D. natronolimnaea* STR1-inoculated plants were successfully colonized by *D. natronolimnaea* STR1 (Supplementary Fig. S3). Scanning electron micrographs confirmed the presence of rhizobacteria on the roots of STR1 inoculated wheat plants (Supplementary Fig. S3).

### Effect of *Dietzia natronolimnaea* strain STR1 inoculation on wheat growth

**Figure 1:**
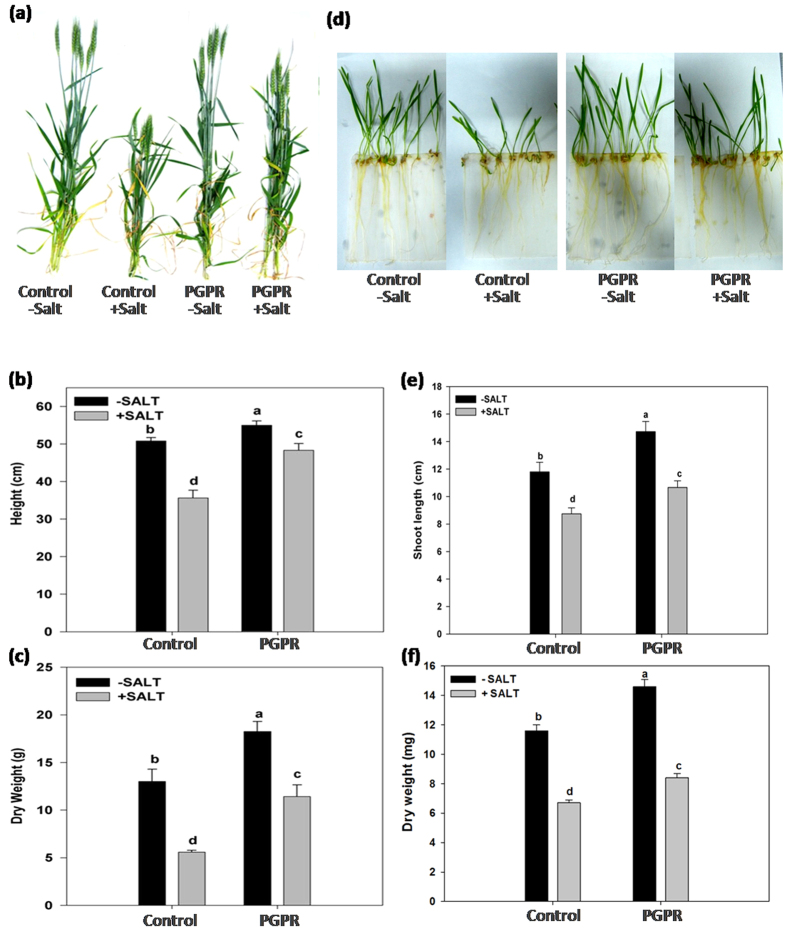
Effect of PGPR inoculation on wheat plant growth under non-saline and saline conditions. (**a–c**) Plants were grown in soil under glasshouse conditions and saline plants supplemented with 150 mM NaCl via irrigation and harvested 60 d after germination. Dry weight and plant height of wheat shoots were measured. (**d–f**) 12 d old wheat plants grown hydroponically in Hoagland nutrient solution. NaCl was added to the nutrient solution to obtain a final concentration of 100 mM. Dry weight and height of wheat shoots were measured after 12 d of germination. Control: without bacterial treatment; PGPR: plants inoculated with *Dietzia natronolimnaea* STR1 strain. Values are mean of five replicates ± standard error of means. Different letters indicate statistically significant differences between treatments (Duncan’s multiple range test *P* < 0.05).

The influence of STR1-inoculation on wheat growth was studied in both saline and non-saline environment. A significant increase in growth of STR1-inoculated wheat was found as compared to uninoculated (control) wheat under both non-saline and saline conditions (Fig. 1). In the case of wheat plants grown in soil, a 57% reduction in dry weight was observed in uninoculated salt-stressed plants in comparison to the non-saline control. PGPR applied plants recorded improved growth in terms of dry weight and plant height (Fig. 1). A marked effect of STR1 inoculation, as well as salinity stress on growth of hydroponically grown wheat plants, was observed (Fig. 1). STR1-inoculated plants recorded higher biomass, shoot and root elongation compare to un-inoculated plants under both non-saline and saline conditions (Fig. 1 and Supplementary Table S1).

### Effect on photosynthetic pigments and biochemical markers

**Figure 2:**
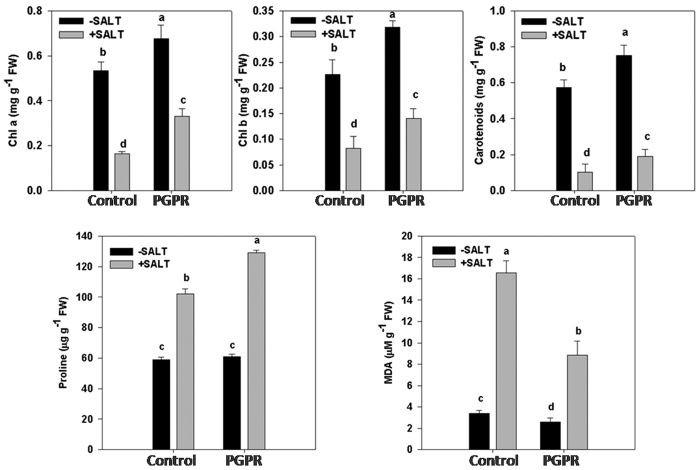
Effect of salt stress on photosynthetic pigments, proline and MDA content of PGPR-inoculated wheat plants. Chlorophyll *a*, Chlorophyll *b*, carotenoid, proline and MDA content was measured in leaves of 60 d old wheat plants grown in soil supplemented with 150 mM NaCl and inoculated with STR1. Control: non-inoculated wheat plants, PGPR: plants inoculated with *Dietzia natronolimnaea* STR1. Values are mean of five replicates ± standard error of means. Different letters indicate statistically significant differences between treatments (Duncan’s multiple range test *P* < 0.05).

A significant reduction in photosynthetic pigments (chlorophyll *a*, chlorophyll *b* and carotenoids) was observed in plants subjected to salt stress irrespective of the bacterial treatments (Fig. 2). PGPR inoculated plants showed increased photosynthetic pigments in comparison to control plants under both saline and non-saline conditions.

There was no significant change in the proline, and MDA (malondialdehyde) contents among un-inoculated as well as PGPR inoculated plants under non-saline conditions, whereas, an increase was registered when plants were subjected to salt stress (Fig. 2). However, a significant increase was observed in PGPR-inoculated plants compared to un-inoculated control except for the MDA content which was found to reduce in PGPR applied plants (Fig. 2).

### Effect on antioxidant enzyme status

The catalase and ascorbate peroxidase levels were similar in both un-inoculated control and rhizobacteria inoculated plants under non-saline conditions (Supplementary Fig. S4). Application of salt stress increased the enzyme levels irrespective of the microbiological treatments; however, the PGPR-inoculated plants recorded significantly higher enzyme content in comparison to control (uninoculated) plants.

### Effect on expression of salt stress responsive genes

Hydroponically grown wheat seedlings were used to study the expression of salt stress-responsive genes. A total of 17 genes (Supplementary Table S3) were analyzed for their change in expression under salinity stress and PGPR inoculation in both shoot and root.

### Expression of genes related to Abscisic acid metabolism

**Figure 3:**
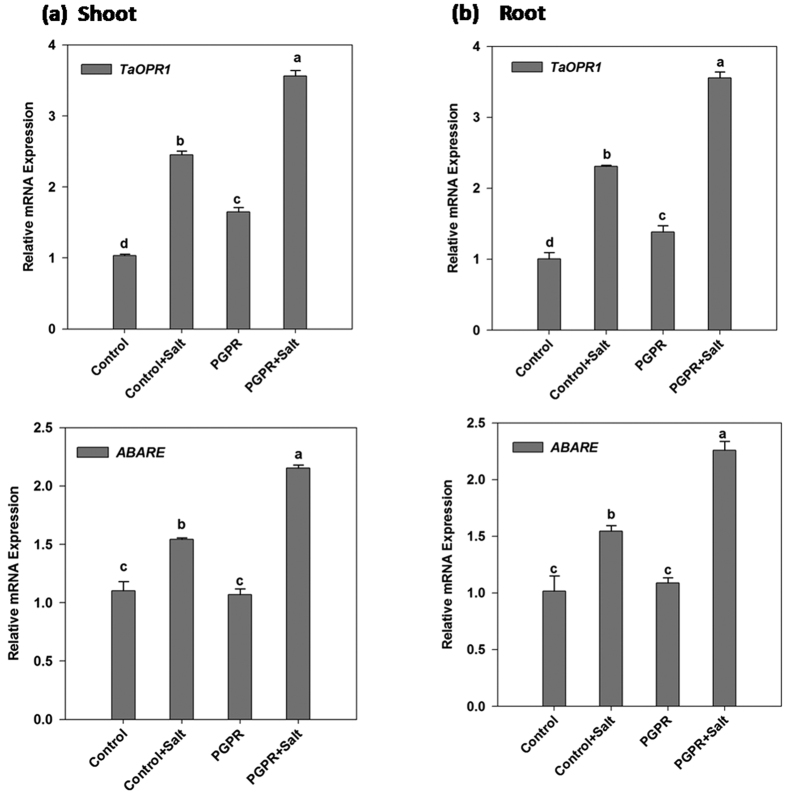
Real time expression analysis of *TaOPR1* and *ABARE* in shoot and root of PGPR-inoculated wheat plants subjected to salt stress. The expression analysis of *TaOPR1* and *ABARE* transcript in (**a**) shoot and (**b**) root of PGPR *Dietzia natronolimnaea* STR1 inoculated 12 d old wheat plants under both non-saline and saline conditions. Un-inoculated wheat plants grown in non-saline condition were used as a control. The data represented means of triplicate biological and experimental repeats; error bars represented SEM. Different letters indicate statistically significant differences between treatments (Duncan’s multiple range test *P* < 0.05).

The effect of salt stress and PGPR inoculation in wheat plants on abscisic acid (ABA) metabolism was studied through the change in gene expression patterns of an ABA-responsive gene (*ABARE*) and *TaOPR1* gene (Fig. 3).

The expression pattern of ABA-responsive gene and *TaOPR1* in both wheat shoot and root were similar. The expression levels in control and PGPR inoculated plants showed minor differences under non-saline conditions, however, higher expression was observed in salt-stressed plants. PGPR-inoculated plants recorded its maximum increase in both root and shoot in comparison to non-saline control plants.

### Expression of genes related to Salt Overly Sensitive (SOS) pathway

**Figure 4:**
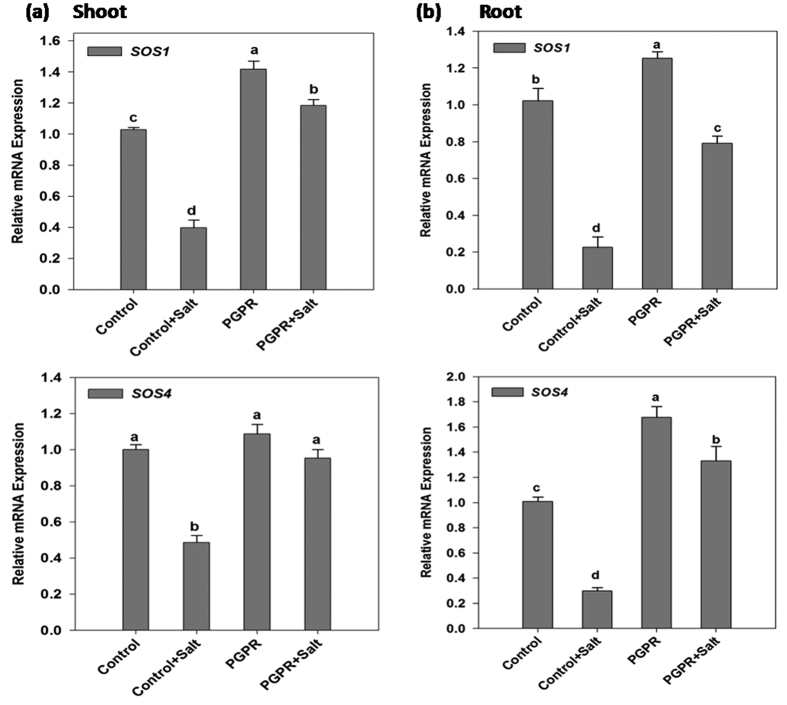
Real time expression analysis of SOS pathway genes viz., *SOS1* and *SOS4* in shoot and root of PGPR-inoculated wheat plants subjected to salt stress. The expression analysis of *SOS1* and *SOS4* transcript in (**a**) shoot and (**b**) root of PGPR *Dietzia natronolimnaea* STR1 inoculated 12 d old wheat plants under both non-saline and saline conditions. Un-inoculated wheat plants grown in non-saline condition were used as a control. The data represented means of triplicate biological and experimental repeats; error bars represented SEM. Different letters indicate statistically significant differences between treatments (Duncan’s multiple range test *P* < 0.05).

The quantitative expression patterns of *SOS1* and *SOS4* genes in shoot and root of wheat plants under salinity stress and PGPR inoculation are shown in Fig. 4.

Analysis of variance for *SOS* pathway genes showed the differential response of shoot under salinity stress as well as PGPR inoculation. PGPR inoculated non-stressed plants recorded a 40% increase in *SOS1* expression in comparison to the uninoculated control plants. Application of salinity stress lowered *SOS1* gene expression in shoots of both uninoculated and PGPR inoculated plants. However, PGPR-inoculated salt-stressed plants recorded 2 fold higher *SOS1* expression in comparison to the uninoculated salt-stressed plants.

The gene expression profile of *SOS1* in roots varied with the application of salinity stress and PGPR inoculation. Similar to the shoot response, PGPR inoculated non-stressed plants recorded the highest expression whereas the salt-stressed plants recorded a decrease in the expression levels. However, among the salt-stressed plants, the PGPR inoculated plants showed 2.6 fold higher *SOS1* expression level in comparison to the uninoculated plants.

*SOS4* expression level in both shoots and roots varied with the treatments. Application of salt stress reduced the expression of *SOS4* in shoots of uninoculated control plants. However, salt stress could not affect the expression of *SOS4* in PGPR inoculated shoots significantly.

Under non-saline condition, *SOS4* expression in roots was higher (65%) in PGPR inoculated plants compared to uninoculated control plants. Salt stress reduced the expression of *SOS4* in roots of both uninoculated and PGPR inoculated plants. However PGPR inoculated salt-stressed plants had 3.5 fold higher *SOS4* expression than that of uninoculated salt-stressed plants.

### Transcription factors

**Figure 5:**
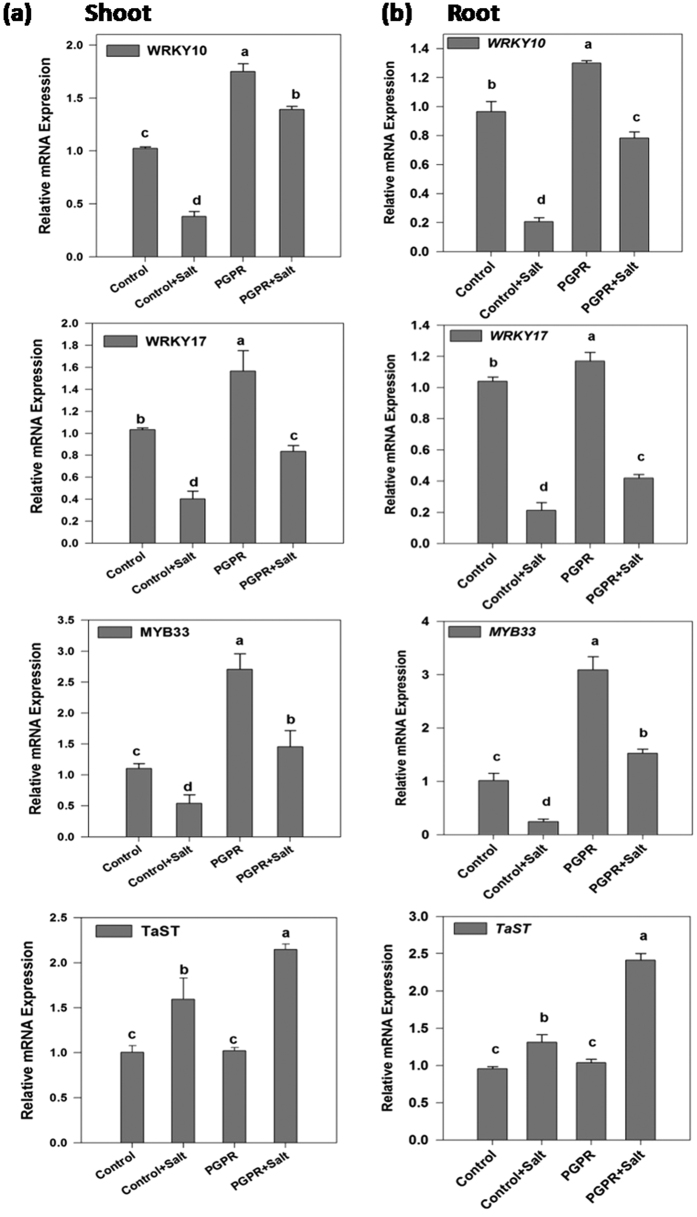
Real time expression analysis of *WRKY*, *MYB* and *TaST* in shoot and root of PGPR-inoculated wheat plants subjected to salt stress. The expression analysis of *WRKY10*, *WRKY17*, *MYB33* and *TaST* transcript in (**a**) shoot and (**b**) root of 12 d old wheat plants inoculated with PGPR *Dietzia natronolimnaea* STR1 under both non-saline and saline conditions. Un-inoculated wheat plants grown in non-saline condition were used as a control. The data represented means of triplicate biological and experimental repeats; error bars represented SEM. Different letters indicate statistically significant differences between treatments (Duncan’s multiple range test *P* < 0.05).

Expression pattern of transcription factors viz., *TaWRKY10*, *TaWRKY17* and *TaMYB33* were examined (Fig. 5). *TaWRKY10* expression level in the non-stressed wheat shoots and roots escalated in the PGPR applied plants as compared to the uninoculated (control) plants. Salt-stressed uninoculated plants recorded a 60 and 80% decrease in *TaWRKY10* expression level in the shoots and roots respectively. However, PGPR-inoculated salt-stressed plants recorded an improvement in the gene expression level as compared to the un-inoculated salt-stressed plants.

*TaWRKY17* expression pattern in shoots and roots was similar to that of *TaWRKY10* across the treatments. PGPR-inoculated plants recorded improved *TaWRKY17* gene expression in comparison to uninoculated (control) plants under both non-saline and saline conditions.

*TaMYB33* expression profile was similar in the wheat shoot and root across the treatments. Among the non-saline plants, the PGPR-inoculated plants recorded approximately 300% (3-fold) increase in the *TaMYB33* expression levels in both shoots and roots. Application of salt stress reduced the *TaMYB33* expression both shoots (50% reduction) and roots (80% reduction) as compared to their respective control plants. The *TaMYB33* expression levels were found to be ameliorated in the PGPR-inoculated salt affected plants in comparison to the control plants.

The expression profile of *TaST* (*T. aestivum* salt-tolerant) gene was similar in the roots and shoots of wheat. No significant difference in the *TaST* expression was observed among the control and PGPR-inoculated non-stressed plants. Salt-stressed control plants recorded 50% higher *TaST* expression in the shoots in comparison to the non-stressed control plants. In the shoot of PGPR-treated salt-stressed plant *TaST* expression was almost 1.5 fold higher than that of salt-stressed control plant (Fig. 5).

### Ion transporters

Quantitative RT-PCR analysis was performed to investigate the transcript levels of genes participating in the ion transport (*TaHKT*, *TaNHX*, *TaHAK*).

**Figure 6:**
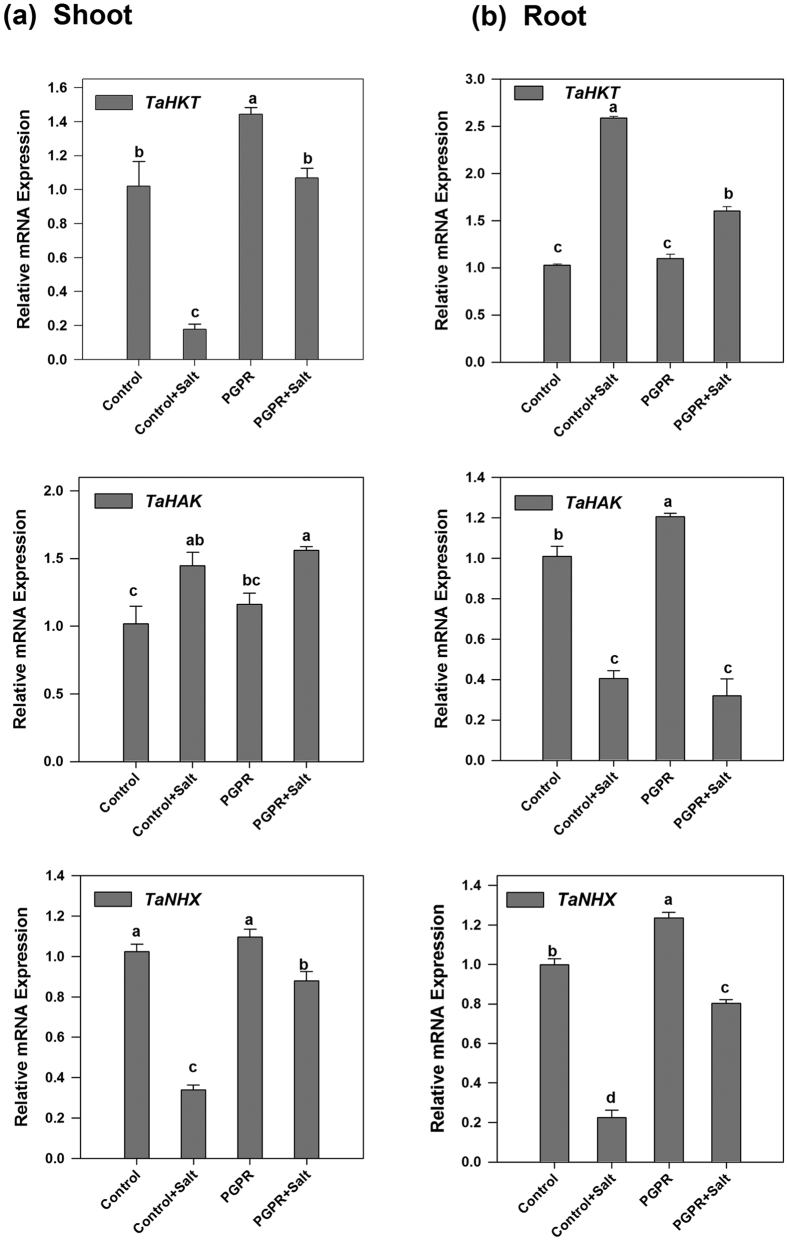
Real time expression analysis of *TaHKT*, *TaHAK* and *TaNHX* in shoot and root of PGPR-inoculated wheat plants subjected to salt stress. The expression analysis of *TaHKT*, *TaHAK* and *TaNHX* transcript in shoot and root of PGPR *Dietzia natronolimnaea* STR1 inoculated 12 d old wheat plants under both non-saline and saline conditions. Un-inoculated wheat plants grown in non-saline condition were used as a control. The data represented means of triplicate biological and experimental repeats; error bars represented SEM. Different letters indicate statistically significant differences between treatments (Duncan’s multiple range test *P* < 0.05).

*TaHKT* expression showed the tissue-specific response in wheat plants. Under non-saline conditions, no significant difference in transcript levels was recorded between un-inoculated and PGPR inoculated plants (both shoot and root). STR1 exposure up- and downregulates *TaHKT* gene expression in shoots and roots respectively. PGPR exposure reduced root *TaHKT* expression by approximately 36% compared with control plants (Fig. 6).

*TaNHX* expression was highest in the PGPR-inoculated non-stressed plants in both shoots and roots. Salt-stressed control plants recorded 80% decrease in the expression levels in shoots as well as roots. PGPR-inoculated salt-stressed plants showed enhanced gene expression as compared to that of uninoculated salt-stressed plants (Fig. 6).

Contrary to *TaNHX* expression profile, *TaHAK* expression was higher in shoots and lower in roots of salt-stressed wheat plants in comparison to their non-stressed counterparts. Maximum *TaHAK* expression was observed in shoots of PGPR-inoculated salt-affected plants. The expression levels of *TaHAK* in roots were similar for uninoculated as well as PGPR-inoculated salt-stressed plants (Fig. 6).

### Antioxidant enzymes

**Figure 7:**
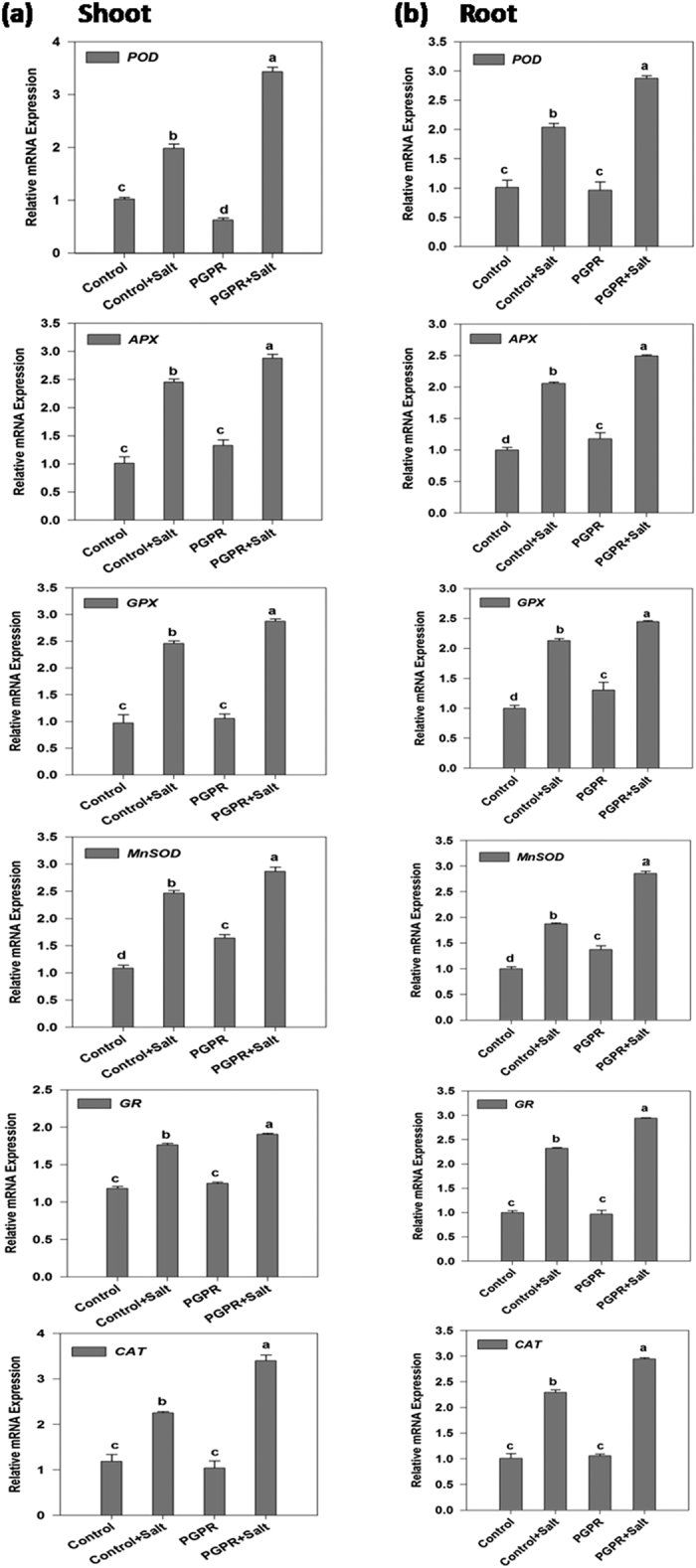
Real time expression analysis of *POD*, *APX*, *GPX*, *MnSOD*, *GR* and *CAT* in shoot and root of PGPR-inoculated wheat plants subjected to salt stress. The expression analysis of *POD*, *APX*, *GPX*, *MnSOD*, *GR* and *CAT* transcript in (**a**) shoot and (**b**) root of 12 d old wheat plants inoculated with PGPR *Dietzia natronolimnaea* STR1 under both non-saline and saline conditions. Un-inoculated wheat plants grown in non-saline condition were used as a control. The data represented means of triplicate biological and experimental repeats; error bars represented SEM. Different letters indicate statistically significant differences between treatments (Duncan’s multiple range test *P* < 0.05).

To evaluate the effect of salt stress treatments on the antioxidant capacity of PGPR-inoculated plants the gene expression patterns of antioxidant enzymes (POD, CAT, APX, GR, GPX, MnSOD) were analyzed using quantitative RT-PCR (Fig. 7).

The expression profile of the antioxidant enzymes followed a similar pattern in wheat roots and shoots. The gene expression patterns for all the antioxidants were similar for uninoculated (control) and PGPR-inoculated salt-stressed plants. The gene expression levels were found to be increased (1–2.5 folds) in the control plants when exposed to salinity. The salt-stressed plants applied with the halotolerant PGPR showed an improvement in the gene expression levels as compared to uninoculated salt-stressed plants. Shoot *APX*, *CAT*, and *POD* of PGPR-inoculated salt-stressed plants recorded a 3-fold increase in the gene expression as compared to uninoculated non-stressed plants. Roots of PGPR-inoculated salt-stressed plants recorded a 2–3 fold increase in the gene expression of the antioxidants in comparison with the non-stressed control plants.

## Discussion

Rhizobacteria have been well documented to promote plant growth as well as alleviate various abiotic stresses including salinity^16^. *Dietzia natronolimnaea* has been extensively exploited for carotenoids production^17^ and bioremediation of hydrocarbons^18^. *Dietzia* species have also been reported to be associated with plants as endophytes^19^ as well as with plant rhizomes growing in soda lakes^20^; however, limited information on their role in plant growth promotion exists^15^. The present study is an effort to identify key genes and transcription factors involved in halotolerant rhizobacteria *Dietzia natronolimnaea* STR1 mediated salt tolerance of wheat plants. Our results suggested that *D. natronolimnaea* STR1 promotes the growth of plants in control conditions as well as under salt stress as illustrated by improved dry weight of wheat plants in both glasshouse and hydroponics. STR1 inoculation affected the root architecture of wheat plant as observed by increased root length under both non-saline and saline conditions. Previous reports^21^ have suggested varying response of rhizobacteria on plant root architecture indicating the specificity of plant-rhizobacterial associations. A recent report^22^ suggests salt-induced burst in ABA production is correlated with organ-specific carotenoid biosynthesis pathway. The plants inoculated with *D. natronolimnaea* STR1 recorded higher proline and lower malondialdehyde (MDA) levels under salt-stressed conditions in comparison to the un-inoculated salt affected plants. MDA content, a result of lipid peroxidation, is a marker of the extent of membrane damage owed to oxidative damage due to the salt stress. Reduced MDA content during salt stress in the PGPR inoculated plants matched with the earlier observations correlating reduced lipid peroxidation to better stress tolerance mechanisms^10^.

The issue of host plant specificity has been well studied in parasitic and mutualistic interactions; however, the question of whether PGPR efficiently interacts only with a specific host remains scantily conversed. Contrary to the popular theory, few reports have suggested strain-specific molecular responses of plants to rhizobacterial inoculation^23^. Rhizobacteria-mediated plant growth is a multigenic process which may be specific to the participating rhizobacteria and plant species^24^. The study was hence, conducted to identify the changes in the expression patterns of genes participating in inherent salt tolerance when inoculated with PGPR under non-stressed as well as salt-stressed conditions. The results of the study support the “additive hypothesis”^12^, suggesting plant growth as affected by bacterial inoculation under salinity stress is a complex phenomenon and involves a cumulative effect of changes in various genes in the global plant metabolic pathways.

Salt stress in plants is a cumulative effect of osmotic and ionic stress which negatively affects the plant growth and yield. Multiple genes are involved in salt tolerance mechanism. A plethora of stress related genes conferring salt stress tolerance in plants have been reported to be involved in signal transduction, ion transporters, transcription regulation and metabolic pathways^25^,^26^. Osmotic stress can be alleviated through ABA-dependent and ABA-independent pathways whereas ionic stress is mitigated through the participation of Salt Overly Sensitive (SOS) pathway and ion transporters via ion homeostasis.

A definite signaling pathway required for the control of ionic homeostasis in plants is the SOS signaling pathway which is a key mechanism for exclusion of Na^+^ and ion homeostasis control at cellular level^27^. SOS1 is one of the many plasma membrane Na^+^/H^+^ antiporters involved in salt tolerance and is crucial for regulating the long distance Na^+^ transport from root to shoot. *SOS4* encodes a pyridoxal kinase, involved in the biosynthesis of pyridoxal-5-phosphate, which acts as an essential cofactor for numerous cellular enzymes. SOS4 regulates Na^+^ and K^+^ homeostasis by modulating the activities of ion transporters. It was also suggested that SOS4 plays a precise role in root hair development in *Arabidopsis*^28^. The results of the present study indicate a decrease in expression levels of *TaSOS1* and *TaSOS4* in shoots and roots under salinity stress which is in contrast with its expression pattern in root reported previously^29^. This could be due to the effect of long-term exposure (10 d) to salt stress in the present experiment whereas Ramezani *et al*. 2012^29^ reported *TaSOS1* and *TaSOS4* expression up to the 72 h of salt exposure. The PGPR-inoculated plants always had higher *SOS* genes expression under salt stress compared to salt stressed un-inoculated plants, suggesting their participation in salt tolerance mechanisms. *SOS4* gene is associated with auxin and ethylene mechanisms as well as the development of root hair in *Arabidopsis*^30^. The improved *SOS4* expression in PGPR inoculated wheat roots can thus be correlated with improvement in root length and overall plant growth in salt-stressed plants.

WRKY TFs play crucial roles in the regulation of many stress induced reactions in plants but until recently unravelling their roles in various abiotic stress responses has lagged behind that of biotic stresses^31^. *TaWRKY10* has been reported to confer salt and drought stress tolerance in transgenic tobacco by regulating the expression of stress-related genes, thus protecting plants from damage^32^. In the present study, *TaWRKY10* expression was found to be reduced in the salt-stressed plants. However, PGPR-inoculated salt-stressed plants recorded increased *TaWRKY10* expression. Salinity and drought stress have been reported to upregulate *TaWRKY17* expression in plants^33^. The expression of *TaWRKY10*, *TaWRKY17* was downregulated under the influence of salinity stress, might be due to long term exposure to salt stress. PGPR-inoculated plants had higher *TaWRKY10*, *TaWRKY17* expression in saline condition compare to un-inoculated plants might be a reason to provide tolerance effect under salt stress. The results indicate a positive effect of PGPR inoculation on the expression levels of transcription factors (TFs), hence improved salinity tolerance. WRKY TFs play the important roles in regulation of water/drought-stress by modulating the cellular osmotic balance, ROS scavenging mechanism and expression of different stress-related genes^34^ as evident by the enhanced and improved growth in PGPR applied plants under glasshouse conditions. Plant growth inhibiting rhizobacteria have been found to induce the response of *WRKY18* genes in *Arabidopsis* demonstrating the direct plant-microbe interaction effects at gene transcript levels^35^.

MYB proteins are one of the main factors for regulatory networks controlling development, metabolism and responses to abiotic and biotic stresses^36^. *TaMYB33* expression has been found to be upregulated during drought, salt and ABA treatments. The ectopic over-expression of *TaMYB33* in *Arabidopsis* enhanced tolerance to salt and drought stresses with no growth inhibition, indicates that the *TaMYB33* is a promising gene candidate for genetic manipulation studies in wheat for providing tolerance to abiotic stress^37^. In the present study because of the long term exposure to salt stress the *TaMYB33* expression was reduced in both root and shoot whereas STR1 inoculated plants had higher *TaMYB33* transcript compared to un-inoculated plant during salt stress probably led to increased salt tolerance. Previously reported that overexpression of *MYB* genes in rice resulted increase in soluble sugars and proline accumulation due to upregulation of proline synthesis and transport related genes, and less accumulation of H_2_O_2_ and MDA under salt stress^38^, suggesting a probable reason for higher proline and reduced MDA levels in rhizobacteria inoculated salt-stressed plants as they had higher *TaMYB* expression compared to un-inoculated salt stressed plant. It has been found that both the SOS pathway^39^ and the SOS-independent pathway^40^ are involved in the perception of the salt stress signal, ionic absorption and intracellular ion compartmentation, however, the involvement of *TaMYB33* is a question yet to be answered. A putative MYB like transcription factor (*LjMAMI*) has been observed to be induced during the mycorrhization process^41^ indicating the influence of plant growth promoting micro-organism on the gene regulatory system of plants. Hence, it can be concluded that the plant-rhizobacteria interactions are governed by the specificity of interactions at metabolic as well as molecular paradigms.

Ion transporters are known as terminal determinants of ionic homeostasis under salt- stressed conditions. Over-expression of *AtNHX1* has been correlated with improved salinity tolerance^42^. Likewise, in the present study, higher expression of *TaNHX* in PGPR-inoculated plants was observed in both shoot and roots in comparison to their un-inoculated counterparts suggesting a likely role of halotolerant rhizobacteria in modulating ion transport mechanisms. SOS1 has been associated with Na^+^/H^+^ antiporters^5^ indicating complex and intertwined mechanisms responsible for enhanced salinity tolerance in plants. Involvement of HKT transporters in salt tolerance mechanism has been reported in both dicot and monocot crops^43^. Hyperaccumulation of Na^+^ in shoots and lower Na^+^ in roots have been observed in *AtHKT1* mutants indicating a direct role of the gene in Na^+^ movement across plant tissues. The *TaHKT1* expression in the present study has been observed to be considerably higher in PGPR-inoculated plants under both non-saline and saline conditions which could be attributed to improved growth in bacterized plants in comparison to control plants. In congruence with the present study, the tissue-specific response of *HKT1* in *A. thaliana* inoculated with *Bacillus subtilis* GB03 has been earlier reported to enhance salt tolerance^44^. HAK type transporters are multigene family members in *Arabidopsis*, common ice plant and barley, and the various genes show specific type of expression patterns in different tissues of the plants^45^. Higher *TaHAK* expression levels can be correlated with improved salinity tolerance in wheat plants. Owing to the tissue-specific response of *HAK* expression, the transcript levels, unlike other transporters, varied in wheat roots and shoots. *TaST* is reported to be a salt-inducible gene and reduce intracellular Na^+^ concentration, increase K^+^ content, and maintain a high K^+^/Na^+^ ratio in the transgenic *Arabidopsis*^46^. *TaST* expression in roots was higher in STR1 treated plants indicating the role of rhizobacteria in triggering the *TaST* expression hence enhanced salt tolerance. Pathogen invasion, wounding, and oxidative stress and events associated with ROS acceleration have been documented to up-regulate *OPR1* genes and have been suggested to be associated with antioxidant activities mediating stress tolerance^47^. It was observed that *TaOPR* responds to salinity stress in ABA-dependent manner and its overexpression improve salinity tolerance in *Arabidopsis* and wheat. Hence, higher *TaOPR1* expression levels can be correlated with enhanced antioxidant response.

Application of abiotic stresses causes an increase in cellular level of ROS like superoxide radical (O^2−^), hydroxyl radicals (OH) and hydrogen peroxide (H_2_O_2_), leading to lipid peroxidation of membranes^48^. Plants have different antioxidant molecules and enzymes that reduce ROS level and alleviate oxidative stress. In the present study, MDA levels reduced in PGPR-inoculated plants, whereas, enzyme activities of CAT and APX in PGPR-inoculated plants growing under NaCl-stress conditions increased significantly when compared with that of the non-inoculated NaCl-stressed plants. The transcript of genes for antioxidative enzymes (*APX*, *CAT*, *POD*, *MnSOD*, *GPX* and *GR*) increased in the PGPR-inoculated plants. Plant tolerance to salinity stress was correlated with the increased expression of *CAT*, *APX*, *MnSOD*, *POD*, *GPX*, and *GR*, suggesting that PGPR triggered the abiotic stress-related defense pathways. Previous reports have demonstrated that plant-associated microorganisms attenuate salt-induced lipid peroxidation as well has higher CAT and APX activities resulting in enhanced salt tolerance^49^. PGPR have also been known to mediate abiotic stress tolerance in plants through modulation of ROS-scavenging enzyme expression^50^.

**Figure 8:**
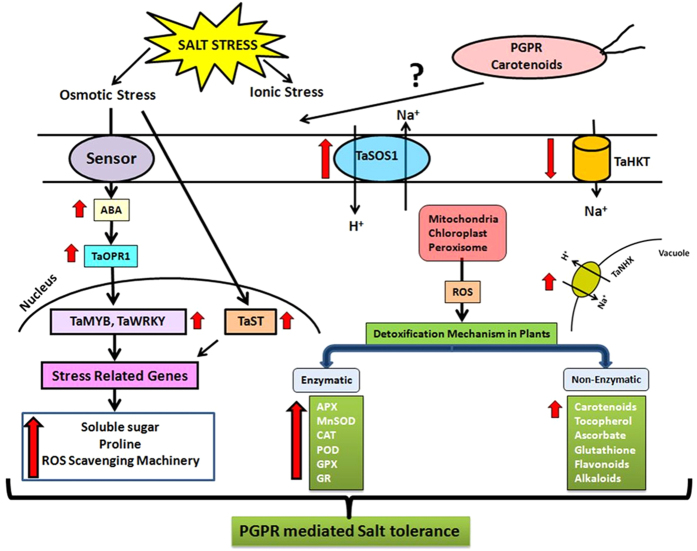
Overview of modulation of expression of genes involved in PGPR *Dietzia natronolimnaea* STR1-mediated salinity tolerance in wheat plants. In the early steps of interaction, *Dietzia natronolimnaea* STR1 triggered signal transduction which modulated the expression of several genes responsible for salt tolerance. Based on the results we propose that carotenoid producing *D. natronolimnaea* STR1 participates in salt tolerance via both ABA-mediated and SOS-mediated pathways by up-regulating the expression of ABA-signalling cascade genes (*TaABARE* and *TaOPR1*), leading to induction of *TaMYB* and *TaWRKY* expression followed by stimulation of expression of a plethora of stress related genes including *TaST*, a salt stress-induced gene, associated with promoting salinity tolerance. Modulation of SOS pathway related genes (*SOS1* and *SOS4*) and tissue specific responses of ion transporters *TaNHX1*, *TaHAK* and *TaHKT1*, were observed in PGPR applied plants. The enhanced gene expression of various antioxidant enzymes such as *APX*, *MnSOD*, *CAT*, *POD*, *GPX* and *GR* and higher proline content in PGPR-treated wheat plants contributed to increased plant tolerance to salinity stress. The red coloured bold arrows indicate the up-regulation or down-regulation of genes.

In conclusion, PGPR *D. natronolimnaea* STR1 promotes growth and protects wheat plants from damage to salt stress. This rhizobacterium appears to confer tolerance to salt stress, at least partly, via modulating the *TaMYB* and *TaWRKY* transcription factors. These transcription factors activate adaptive responses by inducing expression of genes encoding ion channels and transporters to eliminate the accumulation of toxic ions in the cytosol, genes involved in the synthesis of osmoprotectants and antioxidant defense mechanism to reduce reactive oxygen species and MDA contents (Fig. 8). However, further evidences are needed to verify the exact role of these transcription factors along with other putative mechanisms participating in PGPR-mediated salinity tolerance in plants.

## Methods

### Bacterial strain

The bacterial strain *Dietzia natronolimnaea* STR1 (Accession no. KJ413139)^15^ was obtained from the culture collection of Microbial Technology Department, CSIR-CIMAP, Lucknow, India.

### Molecular characterization and phylogenetic analysis of bacteria

Bacterial genomic DNA was isolated using the standard procedures of Chachaty and Saulnier 2000^51^. The extracted DNA was quantified using NanoDrop 1000 spectrophotometer (Thermo Fisher Scientific). Primers used for PCR amplification of the 16S rRNA are described in Supplementary Table S2. For 16S rRNA gene amplification, ~25 ng of genomic DNA and 10 μM of forward (fD1) and reverse (rP2) primers, 0.2 mM of each dNTPs, 0.6 U of Taq DNA polymerase (Bangalore Genei, India), 1X PCR buffer were used for amplification in a Master cycler gradient PCR system (Eppendorf). The thermocycling condition was denaturation at 94 °C for 5 min; 34 cycles of 94 ^o^C for 1 min, 57.4 ^o^C for 1 min, 72 ^o^C for 2 min and a final extension step at 72 ^o^C for 10 min. PCR products were visualized on 1.2% agarose gels and purified using PCR Cleanup Gel Extraction kit (Nucleo-pore, Genetix Biotech Asia Pvt. Ltd. India) following the manufacturer’s protocol. Sequencing was performed on a 3130xl Genetic Analyzer (Applied Biosystems, USA). Sequence analysis was carried out using the NCBI BLAST search (http://www.ncbi.nlm.nih.gov/BLAST/) and nearest neighbor sequences were identified and downloaded from the NCBI database. The 16S rRNA gene sequence of STR1 isolate was aligned with those of 29 other bacteria using ClustalW alignment tool (MEGA5)^52^. A phylogenetic tree was prepared using the bootstrapped neighbor-joining tree method (MEGA5), showed maximum similarity of STR1 with *Dietzia natronolimnaea*.

### Functional traits of STR1 strain

Various plant growth promoting properties were tested in STR1 bacterial strain. The phosphate solubilization test was done on Pikovskaya (PVK) medium^53^. ACC deaminase activities and indole acetic acid (IAA) production were tested using Dworkin and Foster (DF) minimal salts medium^54^ and Salkowsky’s reagent in tryptophan amended medium^55^ respectively. The presence of exopolysaccharides was tested according to the method of Siddikee *et al*.^56^. The *nifH* gene was amplified from the bacterial DNA using primers described in Supplementary Table S2. PCR amplification was performed in 50 μL reaction volume containing 50 ng DNA, 20 μM each of forward (Pol F) and reverse (Pol R) primers, 0.2 mM of each dNTPs (Sigma, USA), Taq polymerase buffer (1X) and 0.6 U of Taq polymerase enzyme (Sigma, USA). PCR conditions consisted of an initial denaturation at 94 °C for 4 min followed by 30 cycles of denaturation at 94 °C for 1 min, annealing at 55 °C for 1 min and extension at 72 °C for 2 min and a final extension step at 72 °C for 5 min with Master cycler gradient PCR system (Eppendorf). PCR products were analyzed in 1.5% (w/v) agarose (Sigma, USA) gels stained with ethidium bromide and visualized under UV light.

### Plant material, growth conditions, and salt stress treatment

Wheat (*Triticum aestivum* L.) cv. HD 2285, was used in the present study. Seeds of HD 2285 were obtained from Indian Agricultural Research Institute (IARI), Pusa, New Delhi, India. The experiments were performed following completely randomized block design in pots (22 cm top diameter x 12 cm bottom diameter x 17 cm height) containing soil, soil supplemented with NaCl and soil supplemented with NaCl and inoculated with halo-tolerant PGPR (*D. natronolimnaea* STR1). The experiments were conducted with three replicates in a glasshouse and the potential of STR1 to alleviate salinity stress in wheat was evaluated. Seeds were surface sterilized by gently shaking in 3% sodium hypochlorite solution containing 0.1% Triton X-100 for 5 min and rinsed four times for 5 min in sterile deionized water. Sterilized seeds were soaked in sterile distilled water for 2 h before sowing in pots (five seeds per pot). The potting mixture consisted of sterilized soil [sandy loam (Ustifluvent)] having pH 7.16, EC 0.46 dS m^−1^, 4.64 g kg^−1^ organic carbon, 127 kg ha^−1^ available N (alkaline permanganate extractable), 10.7 kg ha^−1^ available P (0.50 M NaHCO_3_ extractable), and 97.5 kg ha^−1^ available K (1 N NH_4_OAc extractable). Soil sterilization was performed by autoclaving at 15 lbs/121 °C for 3 h. Pots were watered with sterile water on a daily basis for the first 2 weeks after seed germination and later NaCl stress was applied by supplementing with NaCl solution with concentration gradually increasing to 150 mM. The NaCl concentrations were applied incrementally by 50 mM every week until final concentrations (150 mM) were reached and maintained till plant harvesting. Non-saline plants were irrigated only with sterile water. These plants (60 d after germination) were used for estimation of growth parameters, photosynthetic pigments, total proline content, lipid peroxidation, and activity of catalase and peroxidase.

Gene expression study was performed on hydroponically grown wheat plants. Seeds were surface sterilized in 3% sodium hypochlorite solution containing 0.1% Triton X-100 for 5 min followed by four times washing with sterile distilled water. After extensive washing, seeds were germinated and grown hydroponically on germination sheet rolls placed in 250 mL glass beakers filled with the one-half strength of Hoagland nutrient solution under cool-white fluorescent light (100 μmoles photon m^−2 ^s^−1^) in 14 h light and 10 h dark photoperiod at 24 ^o^C room temperature. Saline stress was applied from the day of germination and up to 12 d. Hoagland nutrient solution was exchanged every day. Sodium chloride was added to the Hoagland nutrient solution to obtain a final concentration of 100 mM.

### Bacterial inoculation

Bacterial inoculation to the wheat plants grown under glasshouse conditions was done prior to the NaCl stress application. Bacterial inoculum was prepared by growing STR1 strains in nutrient broth with 5% (w/v) NaCl at 28 °C for 24 h in an orbital shaking incubator at 100 rpm till late exponential growth phase. Optical density was measured to attain the uniform population of bacteria [~10^8^ colony forming units (CFU) mL^−1^] in the culture prior to inoculation. Culture was centrifuged at 8,000 × g for 10 min. The pellet obtained was washed with sterile distilled water and then re-suspended in 0.85% saline solution. The bacterial suspension was adjusted to A_600nm_ = 1.0. For control plants only sterilized 0.85% saline was applied^57^.

For hydroponics experiments, bacterial culture was harvested by centrifugation (8,000 × g for 10 min, 10 °C), washed once with 0.85% NaCl and re-suspended in 0.01 M MgSO_4_ (~10^5^ C.F.U. mL^−1^). One mL of bacterial suspension was mixed in 49 mL of plant growth medium (half-strength Hoagland nutrient solution). For mock inoculation, 1 mL of 0.01 M MgSO_4_ solution was added into the plant growth medium. Colonization of bacteria in root of wheat plant was determined by re-isolating the inoculated bacteria (STR1) from washed roots using glass beads followed by plating out serial dilutions on NaCl amended nutrient agar plates.

### Measurement of growth parameters

The soil grown plants were harvested after 60 d of germination, and their plant height (cm) and dry weight were measured. Hydroponically grown wheat seedlings were harvested after 12 d of seed germination and their height and dry weight were measured. For dry weight measurement harvested shoots were dried at 70 °C for 5 d, and their final dry weights were measured.

### Measurement of photosynthetic pigments

Chlorophyll *a*, chlorophyll *b* and carotenoid content were measured in leaves of soil grown wheat plant. Photosynthetic pigments were extracted from fresh leaf samples in 80% acetone following the procedures described previously^10^ and the quantification was done following the procedure described by Porra *et al*.^58^.

### Total proline content

The total proline content in leaves was measured following the procedure described previously^59^ with slight modifications. Leaves (100 mg) were homogenized in 1.5 mL of 3% sulphosalicylic acid, and centrifuged at 10,000 × g for 10 min in a refrigerated centrifuge. 100 μL of supernatant was reacted with 2 mL glacial acetic acid and 2 mL acid ninhydrin for 1 h at 100 °C and then kept on ice to terminate the reaction. The reaction mixture was extracted with toluene (1 mL) and the absorbance of the extract was recorded at 520 nm. Proline content was determined by using the proline standard curve.

### Lipid peroxidation

Amount of lipid peroxidation was determined by estimating the malondialdehyde (MDA) produced by the thiobarbituric acid (TBA) reaction^60^. Fresh leaf tissues were homogenized in a mortar and pestle with ice-cold extraction buffer (1.6% Na_2_HPO_4_. 12 H_2_O and 0.7% of NaH_2_PO_4_. 2 H_2_O) and centrifuged at 14,000 × g for 30 min. The supernatant was used for the estimation of MDA. 4 mL of 0.5% (w/v) TBA solution containing 20% (w/v) TBA was added to 1 mL of supernatant. The mixture was heated at 95 °C for 30 min and then immediately cooled on ice. The absorbance of the mixture was measured at 532 nm and the correction for non-specific absorbance was done by subtracting the absorbance at 600 nm. MDA concentration was determined by its molar extinction coefficient (155 mM^−1 ^cm^−1^).

### Quantitative real-time PCR analysis

Total RNA was extracted from the shoots and roots of hydroponically grown 12 d old wheat seedlings treated with 100 mM NaCl and PGPR using TRI-reagent (Sigma-Aldrich Inc. MO, USA). DNA contamination from total RNA preparation was removed by using RNase-free DNase I (TaKaRa Bio Dalian CO., Ltd). cDNA was synthesized from approximately 3 μg of total RNA using a first strand cDNA synthesis kit (Thermo Scientific). PCR conditions were 10 min at 95 ^o^C, followed by 40 cycles of denaturation at 95 ^o^C for 15 sec each and annealing/extension at 60 ^o^C for 1 min each. Primers used for the relative quantification of biosynthetic gene transcripts are described in Supplementary Table S3. The threshold (Ct) value for each gene was normalized against the Ct for actin from wheat which was used as the constitutive reference transcript. Uninoculated plants growing in non-saline condition were used as the calibrator. Fluorescent signal intensities were recorded and analyzed on a StepOnePlus^TM^ (Applied Biosystems) Real-Time PCR System.

### Scanning electron microscopy

Twelve days old wheat plantlets, grown in Hoagland’s hydroponic system, bacterial cells (*D. natronolimnaea* STR1) collected from mid-log phase and wheat plantlets inoculated with bacteria STR1, were washed with 0.2 M Sorensen’s phosphate buffer two times. Furthermore, each sample was fixed with fixing solution (prepared in 0.2 M Sorensen’s phosphate buffer) containing 4% (v/v) formaldehyde and 2.5% (v/v) glutaraldehyde for 4 h at room temperature. Fixed samples were washed with 0.2 M Sorensen’s phosphate buffer three times (each wash was given for 15 min). In addition, each sample was dehydrated through a graded series of absolute ethanol (20, 40, 60, 80 and 100% respectively). Each sample was dried in a critical point dryer in a CO_2_ atmosphere and examined at 20 kv in a JEOL JXA 8100 scanning electron microscope.

### Statistical analysis

Statistical analysis of data were carried out by applying ANOVA, suitable to completely randomized design (CRD) using the software PASW Statistics 18. Significant differences among different treatments were carried out using Duncan’s multiple range test at a significance level of P ≤ 0.05. Two trials were conducted for the experiment and both had a similar variance value; hence, the data of both experiments were combined for further analysis. The gene expression data were analyzed using Duncan’s Test (P < 0.05).

## Additional Information

**How to cite this article**: Bharti, N. *et al*. Plant growth promoting rhizobacteria *Dietzia natronolimnaea* modulates the expression of stress responsive genes providing protection of wheat from salinity stress. *Sci. Rep.* **6**, 34768; doi: 10.1038/srep34768 (2016).

## Supplementary Material

Supplementary Information
