# Supplementary material for: Plant growth promoting rhizobacteria Dietzia natronolimnaea modulates the expression of stress responsive genes providing protection of wheat from salinity stress
Source: Sci Rep. 2016 Oct 6;6:34768. doi: 10.1038/srep34768 (PMC5052518; doi:10.1038/srep34768)
Supplement: Supplementary Information [file srep34768-s1.pdf]

Supplementary Material

Plant growth promoting rhizobacteria *Dietzia natronolimnaea* modulates the expression of stress responsive genes providing protection of wheat from salinity stress

Nidhi Bharti, Shiv Shanker Pandey, Deepti Barnawal, Vikas Kumar Patel, Alok Kalra

Supplementary Table S1 Effect of STR1 inoculation on fresh root weight, dry weight and root length of wheat plants grown under non-saline and saline conditions

Treatments	Fresh root weight (g)	Dry root weight (g)	Root length (cm)
Control	0.0742^{ab}	0.0190^{b}	16.10^{bc}
Control+Salt	0.0536^{c}	0.0153^{d}	14.67^{c}
STR1	0.0785^{a}	0.0217^{a}	18.67^{a}
STR1+Salt	0.0691^{b}	0.0175^{c}	17.50^{ab}

Wheat plants were grown hydroponically in Hoagland nutrient solution for 12 d. Sodium chloride was added to the nutrient solution to obtain a final concentration of 100 mM . Values are mean of ten replicates ± standard error of means. Means, followed by different letters in the same column are statistically different according to Duncan's multiple range test (P<0.05). Control=without bacterial treatment; STR1 = plants inoculated with *Dietzia natronolimnaea*

Supplementary Table S2 List of primers used for PCR

Gene amplified	Primer name	Sequence	Reference
16S rRNA	fD1	5′-AGAGTTTGATCCTGGCTCAG-3′	Weisburg *et al*. 1991
	rP2	5′-ACGGCTACCTTGTTACGACTT-3′	
nifH	Pol F	5′-TGCGAYCCSAARGCBGACTC-3′	Poly *et al*. 2001
	Pol R	5′-ATSGCCATCATYTCRCCGGA-3′	

Supplementary Table S3 List of primers used for quantitative real time PCR analysis

Gene	Primer	Sequence	Reference
TaWRKY10 (EF368361)	Forward	5′-AGCTCGTCTGTGCAGTGCACTTAT-3′	Li *et al*. 2013
	Reverse	5′-TCGTGTACATGCATCCGTGAGATT-3′	
TaWRKY17 (JK546463)	Forward	5'-GACCAAGCGGCTCAACGAT-3'	Garg *et al*. 2012
	Reverse	5'-GGTGCATATGCTAGCTTG-3'	
TaSOS1 (AY326952)	Forward	5'- GTTGTCGGTGAGGTCGGAGGG -3'	Ramezani *et al*. 2013
	Reverse	5'- TCATCTTCTCCTACCGCCCTGC-3'	
TaSOS4 (AY337321)	Forward	5'-ATCCAGTCCCACACCGTCCA -3'	
	Reverse	5'- GCTGATTGCCATTGAGAACCTGTC-3'	
TaST (EF675609)	Forward	5′-CGCAGGCCGTCGTCATG-3′	Huang *et al*. 2012
	Reverse	5′-GACTGATCCTGCCAGCAAACAC-3'	
POD (X56011)	Forward	5'-CAGCGACCTGCCAGGCTTTA-3'	Jiang *et al*. 2012
	Reverse	5'-GTTGGCCCGGAGAGATGTGG-3'	
MnSOD (GI1622928)	Forward	5'-CAGAGGGTGCTGCTTTACAA-3'	Baek and Skinner 2003
	Reverse	5'-GGTCACAAGAGGGTCCTGAT-3'	
CAT (GI5711144)	Forward	5'-CCATGAGATCAAGGCCATCT-3'	
	Reverse	5'-ATCTTACATGCTCGGCTTGG-3'	
APX (TC22268)	Forward	5'-GCAGCTGCTGAAGGAGAAGT-3'	
	Reverse	5'-CACTGGGGCCACTCACTAAT-3'	
GPX (TC22467)	Forward	5'-CCCCCTGTACAAGTTCCTGA-3'	
	Reverse	5'-GTCAACAACGTGACCCTCCT-3'	
GR (TC84151)	Forward	5'-TGCGTCCCGAAGAAGATACT-3'	
	Reverse	5'-GTTGATGTCCCCGTTGATCT-3'	
*TaNHX**	Forward	5'-ATCTACYTSCTCCCKCCCAT-3'	Saqib *et al*. 2005
	Reverse	5'-AGATAGCYCCAATTGCAAGA-3'	
TaMYB33 (JN584645)	Forward	5'-GTCGTCCGCCACAGATTACTC-3'	Qin *et al*. 2012
	Reverse	5'-CGAACGATTATTGTTCCCTTCAC-3'	
TaOPR1 (JQ409278)	Forward	5'-ACTGCCACGACTCCGACCC-3'	Dong *et al*. 2013
	Reverse	5'-CCGATGCGACCGCCTTG-3'	
Actin (AB181991)	Forward	5'-CGAAACCTTCAGTTGCCCAGCAAT-3'	
	Reverse	5'-ACCATCACCAGAGTCGAGCACAAT-3'	
HAK1 (JF495466)	Forward	5’-ACGCTTACGGGATCTGTGTG-3’	This study#
	Reverse	5’-GAGCCGAACACGACGTAGAA-3’	
hkt1 (U16709)	Forward	5’-ATGGGCCGGGTGAAAAGATT-3’	
	Reverse	5’-TCCAGAAGGGGTGAACATGC-3’	
ABARE (JK546530)	Forward	5’-CGATGATGAACTCCACTCA-3’	Garg *et al*. 2012
	Reverse	5’-ATGATATAGTTCCAAAGG-3’	

*Primers for the *TaNHX* were designed from the highly conserved homologue regions of all available *TaNHX* isoforms (NCBI Accession Nos. AY040245, AY040246 and AY296910) from wheat (Saqib *et al*. 2005).

#Primers were designed using Primer Express Software v2.0 (Applied Biosystems).

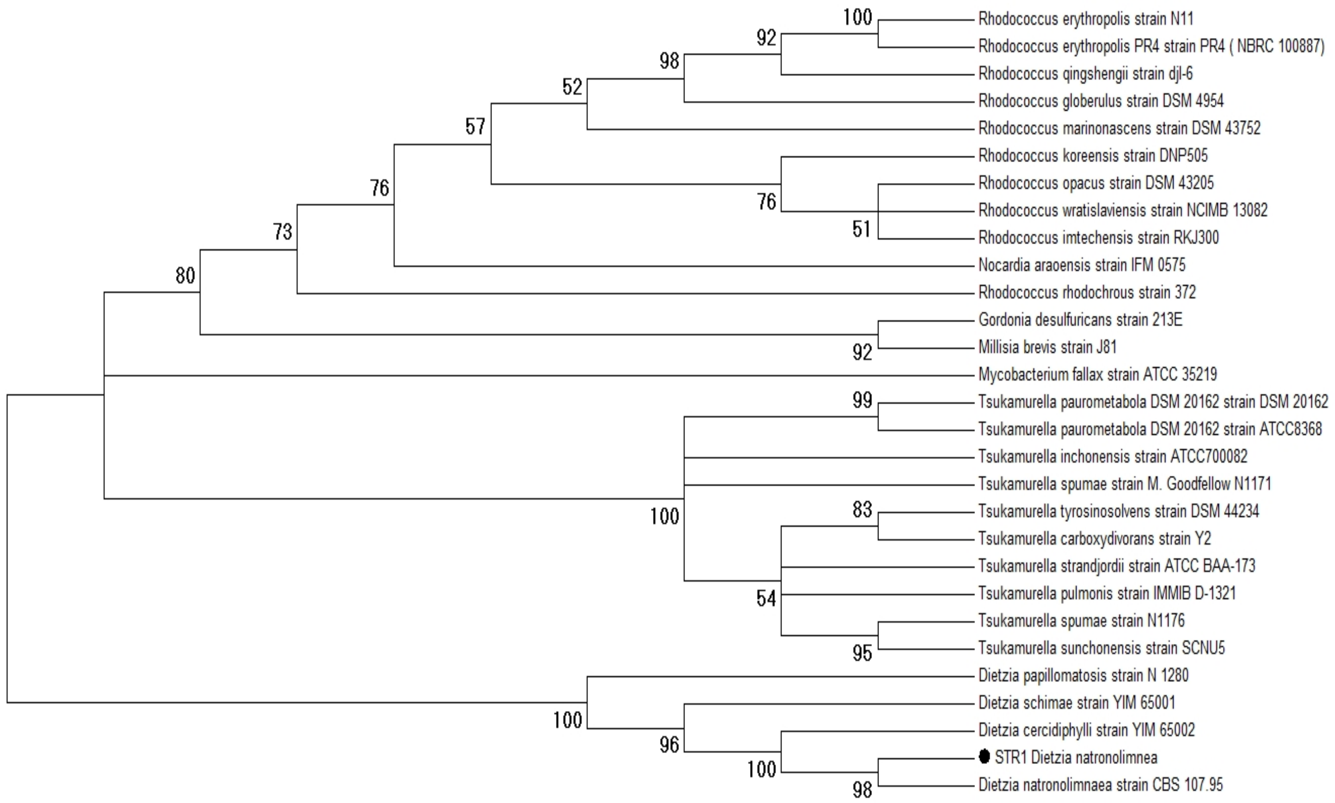

Supplementary Fig. S1: Neighbor-joining tree showing the phylogenetic relationship between *Dietzia natronolimnaea* STR1 and reference strains.

The evolutionary history was inferred using the Neighbor-Joining method. The optimal tree with the sum of branch length = 0.29013879 is shown. The percentage of replicate trees in which the associated taxa clustered together in the bootstrap test (1000 replicates) are shown next to the branches. The tree is drawn to scale, with branch lengths in the same units as those of the evolutionary distances used to infer the phylogenetic tree. The evolutionary distances were computed using the Tamura 3-parameter method and are in the units of the number of base substitutions per site. The analysis involved 29 nucleotide sequences. Codon positions included were 1st+2nd+3rd+Noncoding. All positions containing gaps and missing data were eliminated. There were a total of 1395 positions in the final dataset. Evolutionary analyses were conducted in MEGA5.

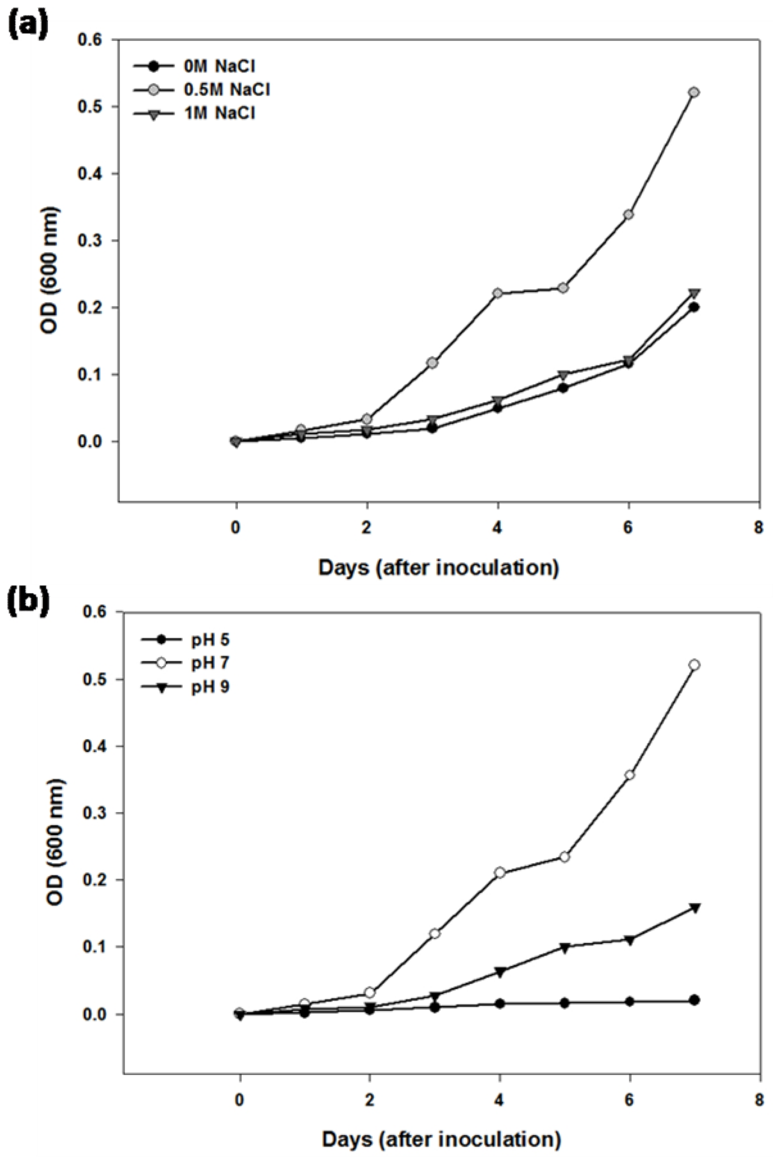

Supplementary Fig. S2: Growth curve for *Dietzia natronolimnaea* STR1 at different conditions. (**a**) Growth pattern at different levels of NaCl viz., 0, 0.5 and 1 M NaCl (at neutral pH). (**b**) Growth pattern at different pH levels viz., 3, 7 and 9.

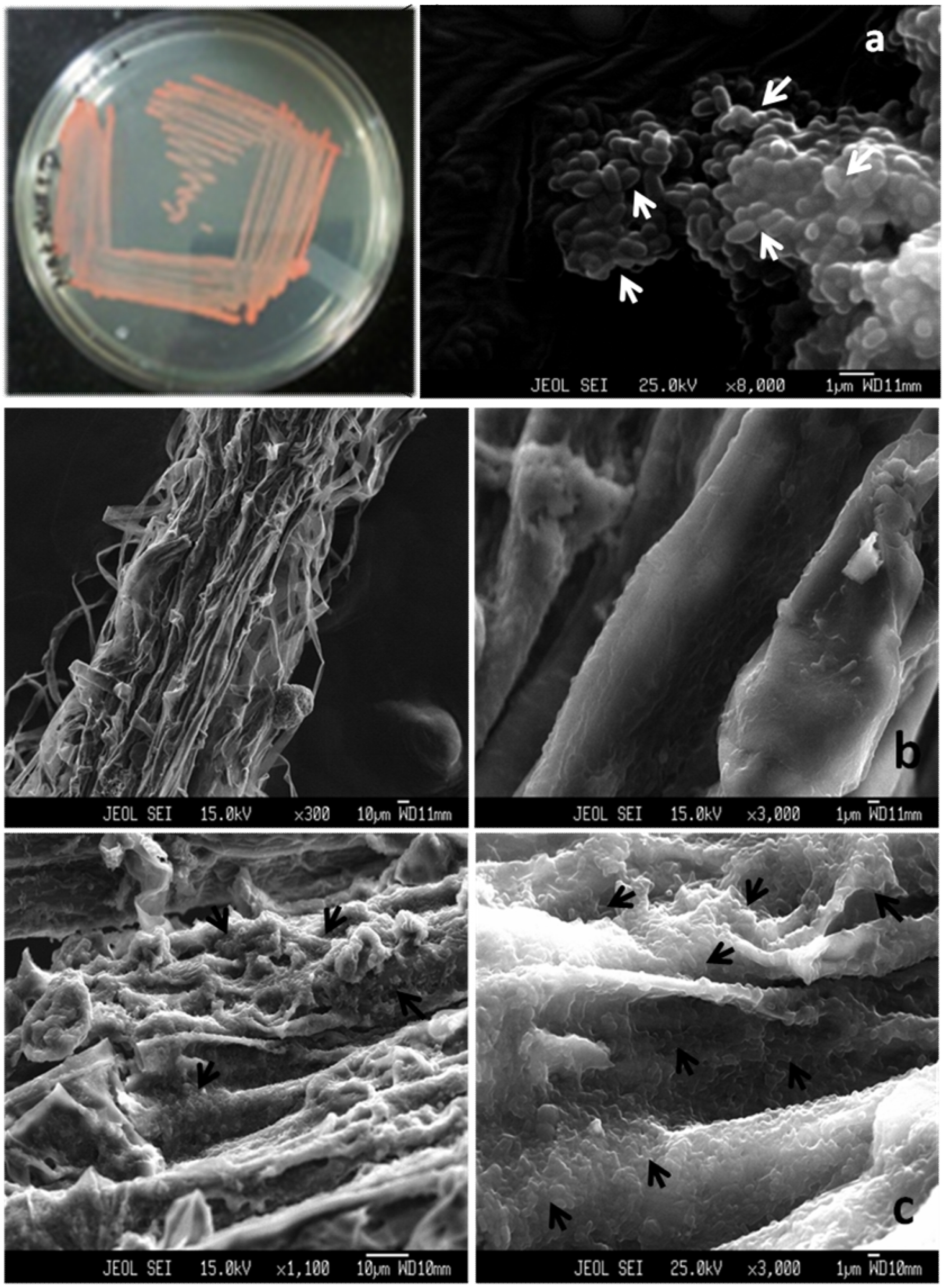

Supplementary Fig. S3: Scanning electron microscopy images showing the colonization of 12 days old wheat seedlings roots by *Dietzia natronolimnaea* STR1. (**a**) pure culture (**b**) un-inoculated roots (**c**) inoculated roots. Arrows indicate bacteria.

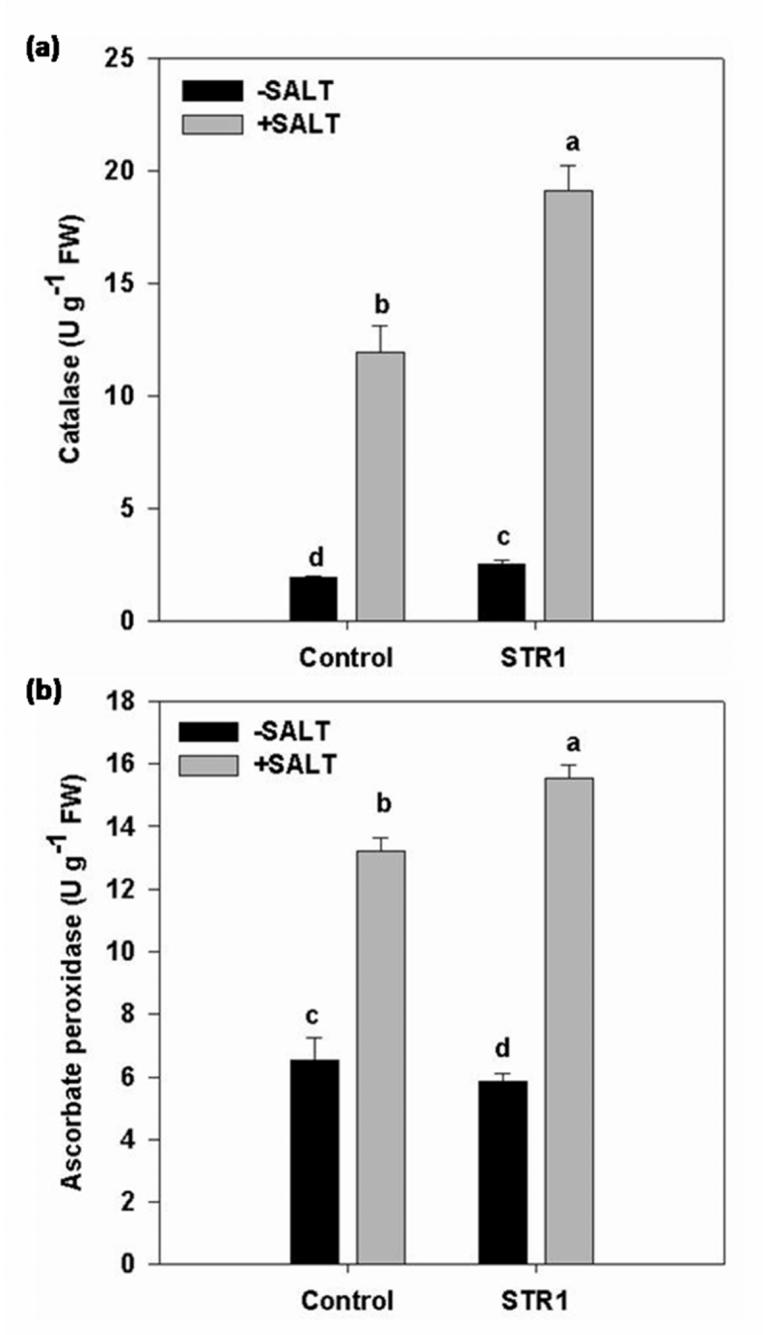

Supplementary Fig. S4: Effect of STR1 inoculation on Catalase and Ascorbate peroxidase activity of wheat plants under non-saline and saline conditions. Catalase and Ascorbate peroxidase activity was measured in leaves of non-inoculated (control) and STR1-inoculated 60 d old wheat plants grown in soil and supplemented with 150 mM NaCl. Control: without bacterial treatment, STR1: plants inoculated with *Dietzia natronolimnaea* STR1. Values are mean of five replicates ±standard error of means. Different letters indicate statistically significant differences between treatments (Duncan's multiple range test $P<0.05$)

Estimation of Catalase activity

Catalase (EC 1.11.1.6) activity was estimated according to Bergmeyer 1970 which measures the initial rate of disappearance of H_2O_2 at 240 nm. 0.5 g tissue was ground in a cold mortar and pestle using liquid nitrogen and suspended in 1.5 mL of homogenization buffer solution (50 mM Tris-HCl, 0.1 mM EDTA, 0.2% TritonX-100, 1 mM PMSF, 2 mM DTT). The suspension was centrifuged at 14,000 rpm for 30 min at 4 ºC. The supernatant was taken for the enzyme assay.

Estimation of Ascorbate Peroxidase (APX) activity

APX (EC 1.11.1.11) activity was measured according to Nakano and Asada 1981. The assay depends on the decrease in absorbance at 290 nm with ascorbate oxidation. 0.5 g tissue, grinded in a cold mortar and pestle with liquid nitrogen and was suspended in 1.5 mL homogenization buffer [50 mM Na-P Buffer (pH 7.0), 2% PVPP, 0.1 mM EDTA, 2 mM ascorbate]. The suspension was centrifuged at 14,000 rpm for 30 min at 4 ºC and the supernatant taken for the enzyme assay.

References

Baek, K. H. & Skinner, D. Z. Alteration of antioxidant enzyme gene expression during cold acclimation of near-isogenic wheat lines. *Plant Science* **165**, 1221-1227 (2003).

Bergmeyer, N. Methoden der enzymatischen, Analyse, vol. 1. Akademie Verlag, Berlin, 636–647 (1970).

Dong, W. *et al*. Wheat oxophytodienoate reductase gene *TaOPR1* confers salinity tolerance via enhancement of abscisic acid signaling and reactive oxygen species scavenging. *Plant Physiol.* **161**, 1217-1228 (2013).

Garg, B., Puranik, S., Misra, S., Tripathi, B. N. & Prasad, M. Transcript profiling identifies novel transcripts with unknown functions as primary response components to osmotic stress in wheat (*Triticum aestivum* L.). *Plant Cell, Tissue and Organ Culture (PCTOC)* **113**, 91-101 (2013).

Huang, X., Wang, G., Shen, Y. & Huang, Z. The wheat gene *TaST* can increase the salt tolerance of transgenic *Arabidopsis*. *Plant Cell Rep.* **31,** 339-347 (2012).

Jiang, L. *et al*. Real-time quantitative PCR monitoring of antioxidant enzyme gene expression in wheat radicles treated with Cu^{2+} and Cd^{2+}. *Life Sci J.* **9**, 1679-1685 (2012).

Li, M. *et al*. Wheat *TaWRKY10-1* is involved in biological responses to the salinity and osmostresses in transgenic *Arabidopsis* plants. *Australian Journal of Crop Science* **7**, 723-729 (2013).

Nakano, Y. & Asada, K. Hydrogen peroxide is scavenged by ascorbate-specific peroxidase in spinach chloroplasts. *Plant Cell Physiol.* **22**, 867–880 (1981).

Poly, F., Ranjard, L., Nazaret, S., Gourbière, F. & Monrozier, L. J. Comparison of *nifH* gene pools in soils and soil microenvironments with contrasting properties. *Appl Environ Microbiol.* **67**, 2255-2262 (2001).

Qin, Y. *et al*. Over-expression of *TaMYB33* encoding a novel wheat MYB transcription factor increases salt and drought tolerance in *Arabidopsis*. *Mol. Biol. Reports* **39**, 7183-7192 (2012).

Ramezani, A. *et al*. Quantitative expression analysis of *TaSOS1* and *TaSOS4* genes in cultivated and wild wheat plants under salt stress. *Mol. Biotechnol.* **53**, 189-197 (2013).

Saqib, M., Zörb, C., Rengel, Z. & Schubert, S., The expression of the endogenous vacuolar Na+/H+ antiporters in roots and shoots correlates positively with the salt resistance of wheat (*Triticum aestivum* L.). *Plant Science* **169**, 959-965 (2005).

Weisburg, W. G., Barns, S. M., Pelletier, D. A. & Lane, D. J. 16S ribosomal DNA amplification for phylogenetic study. *J Bacteriol.* **173**, 697-703 (1991).
